# Prevalence of Multiple Antibiotics Resistant (MAR) *Pseudomonas* Species in the Final Effluents of Three Municipal Wastewater Treatment Facilities in South Africa

**DOI:** 10.3390/ijerph9062092

**Published:** 2012-06-01

**Authors:** Emmanuel E. Odjadjare, Etinosa O. Igbinosa, Raphael Mordi, Bright Igere, Clara L. Igeleke, Anthony I. Okoh

**Affiliations:** 1 Department of Microbiology and Biotechnology, Western Delta University, P.M.B. 10, Oghara, Delta State, Nigeria; Email: ibe22002@yahoo.com; 2 Applied and Environmental Microbiology Research Group, Department of Biochemistry and Microbiology, University of Fort Hare, Private Bag X1314, Alice 5700, Eastern Cape Province, South Africa; Email: eigbinosa@gmail.com (E.O.I.); aokoh@ufh.ac.za (A.I.O.); 3 Department of Microbiology, Ambrose Alli University, P.M.B. 14, Ekpoma, Edo State, Nigeria; 4 Department of Basic Sciences, Benson Idahosa University, P.M.B. 1100, Benin City, Edo State, Nigeria; Email: raphael_mordi@yahoo.com (R.M.); cigeleke@yahoo.com (C.L.I.)

**Keywords:** *Pseudomonas*, antibiogram, multiple-antibiotic-resistance, wastewater effluent

## Abstract

The final effluents of three (Alice, Dimbaza, and East London) wastewater treatment plants (WWTPs) were evaluated to determine their physicochemical quality and prevalence of multiple antibiotics resistant (MAR) *Pseudomonas* species, between August 2007 and July 2008. The annual mean total *Pseudomonas* count (TPC) was 1.20 × 10^4^ (cfu/100 mL), 1.08 × 10^4^ (cfu/100 mL), and 2.66 × 10^4^ (cfu/100 mL), for the Alice, Dimbaza, and East London WWTPs respectively. The effluents were generally compliant with recommended limits for pH, temperature, TDS, DO, nitrite and nitrate; but fell short of target standards for turbidity, COD, and phosphate. The tested isolates were highly sensitive to gentamicin (100%), ofloxacin (100%), clindamycin (90%), erythromycin (90%) and nitrofurantoin (80%); whereas high resistance was observed against the penicillins (90–100%), rifampin (90%), sulphamethoxazole (90%) and the cephems (70%). MAR index ranged between 0.26 and 0.58. The study demonstrated that MAR *Pseudomonas* species were quite prevalent in the final effluents of WWTPs in South Africa; and this can lead to serious health risk for communities that depend on the effluent-receiving waters for sundry purposes.

## 1. Introduction

Globalization of trade, changing population demographics and changes in treatment technology are driving factors in the emergence of new pathogens, including those associated with the water systems [1]. Majority of these emerging waterborne pathogens belong to the “newly recognized” category; implying that, although the etiologic agent was known for a long time, it was recognized only recently as the cause of waterborne illness [2]. *Pseudomonas* species are prominent members of this category of emerging waterborne pathogens [3]. The Pseudomonads comprises species with ecological, economic and health-related importance [4]. Members of this bacterial group are versatile and able to adapt and colonize a wide variety of ecological environments throughout the World, including water, sewage, soil, plants and animals [5]. Most members of the genus (especially *Pseudomonas aeruginosa*) are opportunistic pathogens often associated with infections of the urinary tract, respiratory system, soft tissue, bone and joint, gastrointestinal infections, dermatitis, bacteremia, and a variety of systemic infections, particularly in patients with severe burns, cancer and AIDS [6]. Although the Pseudomonads are not traditionally recognized as waterborne pathogens, recent reports suggest that water systems are increasingly becoming a preferred interface in the epidemiology of the pathogens. *Pseudomonas* species have been incriminated in a number of waterborne outbreaks including those associated with use of recreational waters [7]; showers, hot tubs and swimming pools [6]; thus making the pathogens of growing public health concern.

The emergence of waterborne *Pseudomonas* pathogens is particularly worrisome to stakeholders in the public health sector for two reasons. First, environmental or non-pathogenic forms of the bacteria may serve as a storehouse of genetic determinants which, if transferred to other bacterial strains, may confer novel virulence capabilities [2]. Secondly, recent studies show that prevalence of multiple antibiotics resistant (MAR) *Pseudomonas* strains is on the increase, whereas few antibacterial agents are being developed in parallel. In the United States D’Agata [8] observed an increase from 1% to 16% in the prevalence of MAR (MAR herewith defined as resistance to at least two classes of antibiotics [9]) *Pseudomonas* species during a 9-year period, and Jung *et al*. [10] noted that whereas only 22% of *P. aeruginosa* isolates were resistant to any anti-pseudomonal agent in 1998, 32% of isolates were resistant to at least three agents by 2002. Despite the rising threat of MAR *Pseudomonas* species, no new classes of drugs have been introduced since the advent of imipenem in the early 1980s, and none are expected to appear for commercial use in the near future [11]. Thus limiting treatment options for pseudomonal infections and consequently endangering the public health.

The importance of *Pseudomonas* species as emerging waterborne pathogens is based primarily on their ability to live in biofilms (mixed bacterial populations adherent to specific surfaces within the water system) which often serves as protective cover for the bacteria against biological, physical, chemical and environmental stresses [12]. Growth within biofilms gives rise to extensive genetic diversity that, in turn, enhances the potential for resistance against disinfectants, antibiotics and environmental stress [13]. This explains why *Pseudomonas* species are increasingly getting entrenched in the water system even after disinfection of water resources. *Pseudomonas* survival of chlorine disinfection was recently reported by Samie *et al*. [14]; while Xi *et al*. [15] corroborated by Huang *et al*. [16] suggested that stress-tolerant bacteria selected by chlorination might be more antibiotic resistant; whereas Shivrastava *et al*. [17] found that suboptimal chlorine treatment of drinking water selected for MAR *Pseudomonas aeruginosa*. Given the significant correlation between effluent quality (microbiological and physicochemical) and that of the receiving waters [18], it would be safe to postulate that the release of chlorinated wastewater effluents containing considerable population of *Pseudomonas* species into receiving surface water bodies portend great danger for the South African public health. This is more so as a significant number (about 80%) of the South African population were reported to depend on these surface water bodies for drinking, domestic, recreational and agricultural purposes [19,20]. Furthermore, South Africa has one of the highest HIV/AIDS prevalence in the world [21]; and the given immunocompromised state of such individuals, could lead to serious but avoidable fatalities when exposed to water supply containing *Pseudomonas* species. 

Whereas a considerable number of studies have been carried out on various pathogens isolated from wastewater effluents in South Africa, there is little or no report in the literature on the prevalence and antibiogram of *Pseudomonas* species isolated from chlorinated municipal wastewater effluents in the republic. Given the prevalence and survival strategy of *Pseudomonas* species in water systems, coupled with their opportunistic nature, it is very likely that these pathogens are present in wastewater effluents in South Africa even after disinfection. It is therefore imperative that the presence of these pathogens in wastewater effluents meant to be discharged into South African waters be monitored in the interest of public health. This study was therefore designed to investigate the prevalence and antibiogram profiles of *Pseudomonas* species isolated from chlorinated effluents of three wastewater treatment plants in the Eastern Cape Province of South Africa.

## 2. Materials and Method

### 2.1. Study Site and Sampling

The study sites were located in the Eastern Cape Province of South Africa. Three wastewater treatment plants (WWTPs) were carefully selected to represent rural (Alice: 32°50′36′′S, 26°55′00′′E), peri-urban (Dimbaza: 32°51′28′′S, 27°35′29′′E) and urban (East London: 32°9′7′′S, 27°8′7′′E) settings. Monthly samples were collected between August 2007 and July 2008 from approximately 1 m below the surface of the final treated (chlorinated) effluent just before it was discharged into the receiving water bodies. Samples were collected in duplicates in sterile one litre Nalgene bottles containing 0.1% sodium thiosulphate (3% solution) and transported in cooler boxes containing ice packs to the laboratory for analyses. Sodium thiosulphate was not included in samples meant for physicochemical analyses. All samples were analyzed within 24 h of sample collection.

### 2.2. Physicochemical Analyses

All field meters and equipment were checked and appropriately calibrated according to the manufacturers’ instructions. pH, temperature, total dissolve solid (TDS), and dissolved oxygen (DO), were all determined on site using the multi-parameter ion specific meter (Hanna-BDH laboratory supplies). Turbidity and free chlorine residual (CR) were also determined on site using a microprocessor turbidity meter (model 2100P, HACH Company) and an ion-specific meter (HI 93711, Hanna Instruments) respectively. The concentrations of orthophosphate (PO_4_^3−^), nitrate (NO_3_^−^), nitrite (NO_2_^−^), and chemical oxygen demand (COD) were determined in the laboratory by the standard photometric method [22] using the spectroquant NOVA 60 photometer (Merck Pty Ltd.). Samples for COD analyses were digested with a thermoreactor model TR 300 (Merck Pty Ltd.) prior to analysis using the spectroquant NOVA 60 photometer. 

### 2.3. Isolation, Enumeration and Identification of Pseudomonas Species

The cultural isolation of *Pseudomonas* species was done according to standard spread plate technique on *Pseudomonas* Isolation Agar (PIA agar) (BD Diagnostic Systems). Briefly, aliquots of appropriately diluted samples were inoculated onto PIA agar and incubated at 35 °C for 18–48 h. Typical *Pseudomonas* colonies appear blue-green on PIA agar plates. Total *Pseudomonas* counts (TPC) were taken, and presumptive isolates were purified and stored on nutrient agar slants at 4 °C for further analyses. The presumptive *Pseudomonas* species were confirmed by standard cultural characteristics and biochemical reactions and using API 20NE (10300, BioMerieux). *Pseudomonas aeruginosa* (ATCC 27853) was used as control. 

### 2.4. Antibiogram Assay

#### 2.4.1. Antimicrobial Agents

Nineteen clinically relevant antibiotics were utilized for the antibiogram test. The paper disc antibiotics were supplied by Mast Diagnostics (Merseyside, UK) and included: ampicillin (30 μg), cefotaxime (30 μg), cephalothin (30 μg), cefepime (30 μg), chloramphenicol (10 μg), clindamycin (2 μg), erythromycin (15 μg), gentamicin (10 μg), minocycline (30 μg), nalidixic acid (30 μg), nitrofurantoin (300 μg), ofloxacin (30 μg), oxacillin (1 μg), penicillin G (10 μg), rifampin (5 μg), sulphamethoxazole (5 μg), tetracycline (30 μg), vancomycin (30 μg), and ampicillin-sulbactam (20 μg).

#### 2.4.2. Antibiotic Susceptibility Test

The antibiotic susceptibility test was performed and interpreted based on the disk diffusion method as described by the Clinical and Laboratory Standard Institute [23] using Mueller Hinton agar plates (Biolab, Merck). MAR index was calculated as described by Blasco *et al*. [24] as follows:

    MAR = a/b

where a = number of antibiotics to which the isolate was resistant; b = total number of antibiotics against which individual isolate was tested.

### 2.5. Statistical Analysis

Calculation of the means was done using Microsoft Excel Office 2007. Correlations (paired T-test) and analysis of variance (one-way ANOVA) were performed using the SPSS 15.0 version for windows program (SPSS Inc.). Correlations and test of significance were considered statistically significant at *p* values of <0.05 or <0.01.

## 3. Results

### 3.1. Physicochemical Analyses

**Figure 1:**
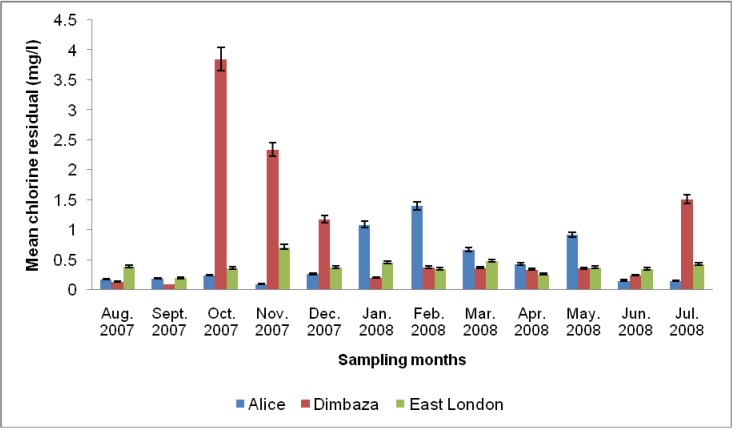
Chlorine residual regime of the final treated effluents from the three wastewater treatment plants sampled.

Table 1 shows the seasonal distribution of some physicochemical parameters across the three wastewater effluents studied. pH and turbidity varied significantly with season (*p* < 0.05) and sampling site (*p* < 0.05); while TDS, nitrate, and phosphate showed significant differences with sampling site (*p* < 0.01) but not with season. Figure 1 shows the free chlorine residual (CR) regime during the study. The CR ranged between 0.097 and 3.85 (mg/L). The highest value was observed in Dimbaza in October 2007; whereas the lowest was observed in Alice in November 2007. The annual average CR values for Alice, Dimbaza and East London were 0.4 mg/L, 0.915 mg/L, and 0.394 mg/L respectively. CR varied significantly with sampling site (*p* < 0.05) but not with season. There was no significant correlation between CR and TPC in Alice and East London treatment plants. However, a significant (*p* < 0.05) negative correlation was observed between CR and TPC at the Dimbaza treatment plant.

**Table 1:** Seasonal distribution of physicochemical parameters of treated wastewater effluents from the three studied plants.

Seasons	Sampling Site	pH	Temperature (°C)	Turbidity (NTU)	^a^ TDS (mg/L)	^b^ DO (mg/L)	^c^ COD (mg/L)	NO_3_^−^ (mg/L)	NO_2_^−^ (mg/L)	PO_4_^3−^ (mg/L)
Spring	^d^ AL	6.8 ± 0.32	20 ± 2.22	3.6 ± 2.10	162 ± 13	4.9 ± 1.52	57 ± 21	11.3 ± 1.0	0.29 ± 0.14	3.50 ± 0.88
	^e^ DMB	7.3 ± 0.44	19 ± 0.74	17.6 ± 15.7	148 ± 9	5.1 ± 0.06	25 ± 8	1.4 ± 0.78	0.1 ± 0.015	0.91 ± 0.15
	^f^ EL	7.1 ± 0.20	20 ± 1.17	12.5 ± 2.53	372 ± 26	5.4 ± 0.13	68 ± 0	3.8 ± 3.21	2.4 ± 3.82	0.38 ± 0.08
Summer	^d^ AL	7.3 ± 1.9	24 ± 3.36	12.2 ± 9	132 ± 10	5.7 ± 4.07	323 ± 457	8.1 ± 2.15	0.21 ± 0.12	1.88 ± 0.53
	^e^ DMB	7.0 ± 0.12	21 ± 1.28	8.2 ± 1.81	128 ± 14	4.9 ± 0.30	330 ± 523	5.9 ± 0.61	0.37 ± 0.09	2.83 ± 0.85
	^f^ EL	7.2 ± 0.19	24 ± 0.95	4.10 ± 1.75	367 ± 80	4.2 ± 0.19	462 ± 599	6.5 ± 0.28	0.21 ± 0.15	0.32 ± 0.13
Autumn	^d^ AL	6.4 ± 0.28	24 ± 1.66	7.39 ± 3	140 ± 8	4.9 ± 0.65	78 ± 59	12.7 ± 5.45	0.13 ± 0.06	1.31 ± 0.91
	^e^ DMB	7.1 ± 0.34	21 ± 2.27	8.7 ± 3.21	115 ± 0.51	4.8 ± 0.34	37 ± 11	5.9 ± 1.17	0.33 ± 0.12	2.97 ± 1.6
	^f^ EL	7.5 ± 0.17	25 ± 1.77	3.8 ± 1.10	470 ± 232	3.9 ± 0.98	48 ± 29	3.4 ± 3.0	0.23 ± 0.05	0.37 ± 0.31
Winter	^d^ AL	6.0 ± 0.55	15 ± 2.02	3.51 ± 1.4	142 ± 9	4.6 ± 1.68	50 ± 31	9.3 ± 6.51	0.22 ± 0.17	1.39 ± 2.15
	^e^ DMB	6.9 ± 0.21	17 ± 2.21	10.5 ± 2.49	117 ± 6	5.3 ± 0.75	78 ± 57	2.2 ± 2.05	0.34 ± 0.28	1.25 ± 2.08
	^f^ EL	6.8 ± 0.10	20 ± 2.03	5.6 ± 0.42	387 ± 17	4.3 ± 0.50	53 ± 31	5.6 ± 1.85	0.73 ± 0.40	0.29 ± 0.13
RecommendedTarget Limits		^g^ 6–9	^g^ ≤25	^g^ 0–1;^h^ ≤5	^g^ 0–450	^i^ ≥5	^j^ 30	^g^ 6; ^j^ 1–5	^g^ 0–6; ^k^ <0.5	^k^ 0.005

Legend: ^a^ Total Dissolved Solids; ^b^ Dissolved Oxygen; ^c^ Chemical Oxygen Demand; ^d^ Alice wastewater treatment plant; ^e^ Dimbaza wastewater treatment plant; ^f^ East London wastewater treatment plant; ^g^ Target limit for domestic water uses in South Africa [25]; ^h^ Target limit for effluent to be discharged into surface waters [26]; ^i^ Target limit for the support of aquatic life [27]; ^j^ Target limit for effluent to be discharged into the environment [28]; ^k^ Target limit that would reduce eutrophication in aquatic ecosystems [29].

### 3.2. Total Pseudomonas Counts (TPC)

**Figure 2:**
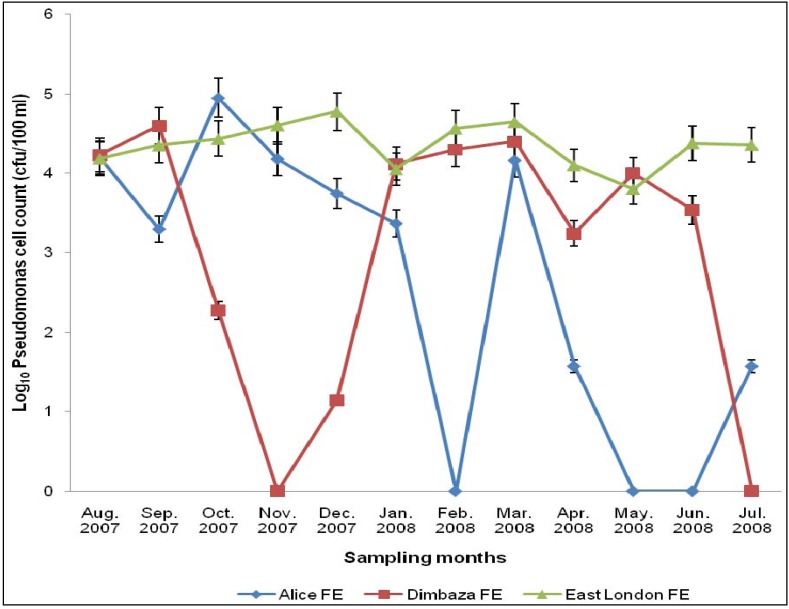
Total *Pseudomonas* count in the treated final effluents.

Figure 2 shows the total *Pseudomonas* counts (TPC) during the study. TPC ranged from 0 to 4.9 × 10^4^ cfu/100 mL. The highest TPC was observed in the Alice plant in October 2007; while the lowest counts were observed in the Alice (February, May and June 2008) and Dimbaza (November 2007 and July 2008) treatment plants. The annual average TPC for Alice, Dimbaza and East London plants were respectively 1.20 × 10^4^ cfu/100 mL, 1.08 × 10^4^ cfu/100 mL and 2.66 × 10^4^ cfu/100 mL. TPC varied significantly (*p* < 0.5) in effluents collected from Alice and East London treatment plants, but not with season. No significant difference was observed for other treatments either with season or sampling site.

### 3.3. Pseudomonas Isolates and Antibiogram

A total of 39 strains of *Pseudomonas* belonging to four species (*P. aeruginosa*, *P. luteola*, *P. mendocina* and *P. fluorescens*) were isolated during this study. Ten of these isolates were randomly selected for the antibiogram assay against the panel of 19 antibiotics (Table 2 and Table 3). The tested isolates showed complete sensitivity to gentamicin (aminoglycosides) and ofloxacin (fluoroquinolones); and high level of sensitivity to clindamycin (lincosamides; 90%), erythromycin (macrolides; 90%) and nitrofurantoin (nitofurantoins; 80%). Intermediate resistance were observed against chloramphenicol (phenicols; 50%), minocycline (tetracyclines; 60%), nalidixic acid (quinolones; 70%), vancomycin (glycopeptides; 60%) and ampicillin-sulbactam (β-Lactams; 50%). All tested isolates exhibited complete to near complete (90–100%) resistance to the penicillins (ampicillin, penicillin G, and oxacillin), rifampin (ansamycins), and sulphamethoxazole (folate pathway inhibitors); and a high (70%) level of resistance to the cephems (cefotaxime, cephalothin, and cefepime). The tested isolates showed a high degree of multiple antibiotics resistances (MAR) ranging between five and 11 antibiotics, distributed among three to seven antibiotic classes (Table 3). 

**Table 2:** Antibiogram of randomly selected *Pseudomonas* isolates from the final effluents of the three wastewater treatment plants.

Antibiotics Class	Antibiotics	Number of isolates (%)		
		Sensitivity	Intermediate	Resistant
Penicillins	Penicillin G	0(0)	0(0)	10(100)
	Ampicillin	0(0)	1(10)	9(90)
	Oxacillin	0(0)	0(0)	10(100)
Cephems	Cefotaxime	1(10)	2(20)	7(70)
	Cefepime	3(30)	0(0)	7(70)
	Cephalothin	0(0)	3(30)	7(70)
Folate Pathway Inhibitors	Sulphamethoxazole	0(0)	1(10)	9(90)
Ansamycins	Rifampin	0(0)	1(10)	9(90)
Quinolones	Nalidixic acid	1(10)	7(70)	2(20)
β-Lactam/β-Lactamase Inhibitor Combinations	^a^ Ampicillin/sulbactam	1(10)	5(50)	3(30)
Phenicols	Chloramphenicol	4(40)	5(50)	1(10)
Tetracyclines	Tetracycline	5(50)	4(40)	1(10)
	Minocycline	2(20)	6(60)	2(20)
Aminoglycosides	Gentamicin	10(100)	0(0)	0(0)
Fluoroquinolones	Ofloxacin	10(100)	0(0)	0(0)
Macrolides	Erythromycin	9(90)	1(10)	0(0)
Glycopeptides	Vancomycin	1(10)	6(60)	3(30)
Nitrofurantoins	Nitrofurantoin	8(80)	1(10)	1(10)
Lincosamides	Clindamycin	9(90)	0(0)	1(10)

^a^ Ampicillin-sulbactam profile was not determined for one isolate; hence 9 were reported.

**Table 3:** Multiple antibiotics resistance (MAR) of the *Pseudomonas* isolates.

Isolates Code	Organism Identity	Antibiotics	MAR Index
Control	*P. aeruginosa* (ATCC 27853)	AMP, CEP, CHL, CLI, MINO, NAL, NIT, OXA, PEN, RIF,SMX, TET, VAN, SAM	0.74
AL 1	*P. aeruginosa*	CEP, CHL, CLI, NIT, OXA, PEN, SMX, VAN	0.42
AL 2	*P. fluorescens*	AMP, CTX, CPM, OXA, PEN, RIF, SMX, SAM	0.42
DB 1	*P. aeruginosa*	AMP, CTX, CEP, CPM, NAL, OXA, PEN, RIF, SMX, TET, VAN	0.58
DB 2	*P. fluorescens*	AMP, CTX, CEP, CPM, OXA, PEN, RIF, SMX	0.42
EL 1	*P. fluorescens*	AMP, CTX, OXA, PEN, RIF	0.26
EL 2	*P. aeruginosa*	AMP, CEP, CPM, NAL, OXA, PEN, SMX, VAN, SAM	0.47
EL 3	*P. fluorescens*	AMP, CTX, CEP, CPM, OXA, PEN, RIF, SMX	0.42
EL 4	*P. aeruginosa*	AMP, MINO, NAL, OXA, PEN, RIF, SMX	0.37
EL 5	*P. aeruginosa*	AMP, CTX, CEP, CPM, OXA, PEN, RIF, SMX, SAM	0.47
EL 6	*P.. fluorescens*	AMP, CTX, CEP, CPM, OXA, PEN, RIF, SMX	0.42

Legend: AMP—Ampicillin; CEP—Cephalothin; CHL—Chloramphenicol; CLI—Clindamycin; MINO—Minocycline; NAL—Nalidixic Acid; NIT—Nitrofurantoin; OXA—Oxacillin; PEN—Penicillin G; RIF—Rifampin; SMX—Sulphamethoxazole; TET—Tetracyclin; VAN—Vancomycin; SAM—Ampicillin-Sulbactam; CTX—Cefotaxime; CPM—Cefepime.

There was no clear pattern of MAR along the lines of isolate origin. The MAR index varied from 0.26 to 0.58 and 0.74 for the control strain. The modal MAR index for the tested isolates was 0.42. 

## 4. Discussion

Values for the physicochemical parameters (especially those of Alice and East London treatment plants) and their potential impact on the receiving environments and public health were reported and discussed in details in our previous studies [30,31,32]. We shall therefore restrict our discussion to the immediate health and environmental impacts of the physicochemical parameters evaluated in this study. The physicochemical quality of the effluents across the three treatment plants were generally compliant to recommended limits for pH, temperature, TDS, DO and nitrite (except for nitrite values recorded at East London in spring and autumn) with respect to effluents meant for domestic uses [25] and those to be discharged into the receiving environment [26,27] in lieu of preserving public health and protecting aquatic life. However, the effluent quality across the three sampled sites generally fell short of target limits for turbidity, COD, and phosphate [25,26,28,29]. Whereas nitrate quality for Dimbaza and East London effluents met the recommended standard for domestic uses, effluent quality at the Alice treatment plant fell short of target limits for this parameter. The observation suggests that effluent emanating from the Alice treatment plant is of poor quality and may compromise public health; especially those of infants and pregnant women [25]. The chlorine residual values generally fell short of the recommended limit (0.3 to 0.6 mg/L) of no risk at point of use [33] and suggest that the effluents may not be appropriate for domestic uses. The observation is particularly significant in view of the high level of turbidity recorded across the sampling sites; which may be indicative of high organic matter content [34] and may result in increased chances of trihalomethane formation in chlorinated effluents [27]. Trihalomethane is a carcinogenic compound formed as a by-product of chlorine and organic matter reaction in water systems and has been reported to have serious health implications for aquatic life and humans exposed to it [35,36]. 

Values for TPC in this study were lower than those (10^4^ to 10^6^ cfu/100 mL) reported previously [37] but similar to the annual average (2.06 × 10^4^) observed by Alaoui *et al*. [9]. Although the free chlorine residual regime across the sampling sites were relatively high, the concentrations were not enough to eliminate *Pseudomonas* species from the effluents (Figure 1 and Figure 2). This ineffectiveness of CR on TPC was generally evident in the lack of significant correlation observed between both parameters; except as seen at the Dimbaza plant where significant (*p* < 0.05) negative correlation between CR and TPC was recorded. The observation suggests that the *Pseudomonas* isolates in this study were generally resistant to chlorination even at concentrations far higher than the recommended limits (0.3–0.6 mg/L) of no risk at point of use [33]. The high level of turbidity observed across all three treatment plants during the study (Table 1) could be a factor in the ineffectiveness of CR on TPC [38]. Turbidity as a measure of suspended particles in water system encourages the growth of bacteria as biofilms which in turn serves as protective cover for the bacterial community against biological, physical, chemical (including chlorination) and environmental stresses [12]. The observation of this study is consistent with the reports of Price and Ahearn [39] who observed isolation of *Pseudomonas* species at CR concentrations as high as 3 mg/L. However, Mena and Gerba [7] reported that although *P. aeruginosa* has a reputation for being resistant to disinfection, most studies show that it does not exhibit any marked resistance to the disinfectants used to treat drinking water such as chlorine, chloramines, ozone, or iodine. The significance of this observation is that operators of WWTPs may be forced to increase their CR dosage; and attempts to eliminate *Pseudomonas* from water supply using relatively high dose of disinfectant may produce disinfection by-products more hazardous than the pathogen itself [40].

The annual average TPC across the three studied WWTPs fell short of the recommended limits (0 cfu/100 mL of faecal coliforms) in lieu of the presence of pathogens in effluents to be discharged into the environment [25]. The observation suggests that all three final effluents were of poor microbial quality throughout the study and thus posed serious health risk to communities that employ the receiving waters for sundry uses. Several disease outbreaks such as cholera, salmonellosis, cryptosporidiosis, and giardiasis, have been linked to wastewater contamination of source waters in South Africa and elsewhere [14]. However, there is little or no report of wastewater-related pseudomonal infections in South Africa. This may partly be due to the fact that *Pseudomonas* species were not usually regarded as waterborne pathogens and as such were not screened for in suspected water samples. This practice creates opportunity for the pathogen to be unaccounted for in relevant water/wastewater samples. And since about 84% of pathogens responsible for waterborne outbreaks in South Africa were reportedly unknown [41], it is possible that waterborne pseudomonal outbreaks occurred without notice in the past. 

Thirty-nine (39) strains of *Pseudomonas* belonging to four species (*P. aeruginosa*, *P. luteola*, *P. fluorescens, and P. mendocina*) were isolated during this study. All four representative species have been reported in bioremediation/biodegradation studies [42,43,44,45] as well as Pseudomonal infections [46,47,48,49], suggesting that municipal wastewater effluent is an important reservoir of *Pseudomonas* species of both environmental and clinical significance. It is difficult to differentiate between pathogenic and non-pathogenic strains of *Pseudomonas* species. According to Alonso *et al*. [42] opportunistic pathogens like *Pseudomonas* species (with broad-range ecological distribution) may not show a clear delineation between virulent and non-virulent strains. 

Consistent with the observation of this study *Pseudomonas* species were reported to be highly sensitive to gentamicin [50] and ofloxacin [47]. The observation is contrary to a previous report suggesting that fluoroquinolones have lost their effectiveness against *P. aeruginosa* strains due to resistance [51]. Contrary to the observation of this study, Navon-Venezia *et al*. [11] reported considerable *Pseudomonas* resistance to the aminoglycosides (including gentamicin) and fluoroquinolones (including ofloxacins) in clinical isolates; while Lateef [52] observed high resistance to both antibiotics in *Pseudomonas* strains isolated from pharmaceutical effluents. The observations were not surprising as clinical and pharmaceutical environments tend to exert more selective pressure (leading to antibiotic resistance) on bacterial populations than non-clinical/non-pharmaceutical (e.g., municipal effluent) environments [53,54,55]. The isolates presented in our work showed high (80–90%) levels of sensitivity to clindamycin, erythromycin and nitrofurantoin (Table 2). Although *Pseudomonas* sensitivity to clindamycin and nitrofurantoin were not common in the literature, Nagata *et al*. [56] reported the macrolides (e.g., erythromycin) to be effective against *P. aeruginosa* biofilm formation; while Navon-Venezia *et al*. [11] suggested that this inhibitory action may explain the salutary effects of the macrolides on *P. aeruginosa*-associated chronic lung diseases, such as cystic fibrosis and diffuse panbronchiolitis. Contrary to the observation of this study, several reports have been documented on *Pseudomonas* resistance to clindamycin [57,58] and nitrofurantoin [58,59,60]. Whereas the tested isolates in this study exhibited intermediate sensitivity to the tetracyclines (tetracycline (40%), minocycline (60%)), nalidixic acid (70%), ampicillin/sulbactam (50%) and chloramphenicol (50%); reports in the literature suggests that *Pseudomonas* species were frequently resistant to these antibiotics [59,60,61,62]. However, Jombo *et al*. [59] reported sensitivity to chloramphenicol by *P. aeruginosa* strains isolated from urinary tract infection patients in Jos, Nigeria; while Lateef [52] observed Pseudomonal sensitivity to tetracyline in isolates from pharmaceutical effluents.

Our tested isolates exhibited high levels of resistance to the penicillins (90–100%) the cephems (80%), rifampin (70%) and sulphamethoxazole (70%) in agreement with reports of other authors [6,63,64]. Conversely, Gad *et al*. [61] reported low resistance (29%) to cefepime, while Cabrera *et al*. [65] observed high sensitivity to the cephems. According to Pirnay *et al*. [6] *Pseudomonas* species were naturally resistant to the penicillins, cephems and rifampin because they have relatively impermeable membrane, inducible efflux systems and a chromosomally encoded inducible β-lactamase. However, Murray *et al*. [66] demonstrated that chlorination of sewage may contribute to bacteria resistance to ampicillin and cephalothin (cefalotin). Although, the mechanism of chlorine-induced antibiotic resistance in bacteria is still unknown, Xi *et al*. [15] suggested the possibility of chlorine disinfection increasing expression of multidrug efflux pumps, resulting in resistance to disinfection by-products as well as antibiotics. 

Although Malekzadeh *et al*. [67] reported *Pseudomonas* isolates that were resistant to only single antibiotics, all the tested isolates in this study showed multiple antibiotic resistances (MARs) ranging from five to 11 antibiotics distributed among three to seven classes. Consistent with the observation of this study, Paul *et al*. [68] reported MAR *Pseudomonas* strains with resistance patterns varying between five and eight antibiotics; while Lateef [52] documented MAR *Pseudomonas* with resistance patterns of two to seven antibiotics. Two major intrinsic mechanisms were reported to confer bacterial resistance to multiple antimicrobial drug classes: mutations in outer membrane porins resulting in reduced permeability to antimicrobials; and over expression of multidrug efflux pumps, which tend to pump out antibiotics before they (the antibiotics) have the opportunity of acting on their target [11,64]. In addition, Navon-Venezia *et al*. [11] observed that MAR bacterial strains may also arise due to unrelated mechanisms accumulating sequentially in an organism. The MAR indices were higher than the 0.2 limit in all our tested isolates (Table 3). The observation indicates that isolates in this study originated from high risks source(s) of contamination where antibiotics are often used [68]. The observation was not surprising as livestock farms were scattered around the immediate catchments of the three WWTPs under study. 

## 5. Conclusions

This study demonstrated that MAR *Pseudomonas* species were prevalent in chlorinated municipal wastewater effluents in South Africa. Since the emergence of MAR *Pseudomonas* species is a public health issue, our data support the need for regular and consistent monitoring of municipal sewage effluents with a view to preventing the dissemination of these pathogens into the environment. 
